# Implications of a novel *Pseudomonas* species on low density polyethylene biodegradation: an *in vitro to in silico* approach

**DOI:** 10.1186/2193-1801-3-497

**Published:** 2014-09-02

**Authors:** Mayuri Bhatia, Amandeep Girdhar, Archana Tiwari, Anuraj Nayarisseri

**Affiliations:** School of Biotechnology, Rajiv Gandhi Technical University, (RGTU, State Technical University of Madhya Pradesh), Airport Bypass Road, Bhopal, 426033 India; In Silico Research Laboratory, Eminent Biosciences, Vijaynagar, Indore, 452010 India

**Keywords:** LDPE, Biodegradation, *Pseudomonas citronellolis* EMBS027, LDPE degrading species

## Abstract

Degradation of Petroleum-plastics like Low Density Polyethylene (LDPE) is a budding challenge due to increasing white pollution. The present investigation has focused the aspect through microbial assisted biodegradation. Various indigenous microorganisms were isolated from collected municipal landfill soil. Growth medium enriched with 0.2 g of LDPE powder was used to screen the soil bacteria with biodegradation potential. The screened bacteria were subjected to biodegradation assay in presence of LDPE sheets in growth medium. Four strains gave 5%, 17.8%, 0.9% and 0.6% degradation rate based on weight loss in the conducted *in vitro* assay for four days. The maximum degraded sheet was analyzed through Scanning Electron Microscopy, Fourier transform infrared spectroscopy and Thermogravimetry, taking undegraded LDPE sheet as control. Results illustrated one-step weight loss with control and three-step weight loss with test. Thus, it proved the efficacy of isolated strain. The strain identification was carried out by genomic DNA isolation followed by PCR and 16S rRNA sequencing. Genotypic identification revealed the bacterium as *Pseudomonas citronellolis.* BLAST gave a similarity with the database of 96%, thus phylogenetic assessment clarified the bacterium as a novel strain. The isolate was named as *Pseudomonas citronellolis* EMBS027 and sequence was deposited as LDPE degrading species, in GenBank with accession number KF361478.

**Electronic supplementary material:**

The online version of this article (doi:10.1186/2193-1801-3-497) contains supplementary material, which is available to authorized users.

## Introduction

Petroleum plastics are the non-biodegradable synthetic polymers that accumulate at the rate of 25 million per year, contaminating the soil and water (Eubeler et al. 2010). Low Density Polyethylene belongs to thermoplastics class (Pramila et al. 2012) and is believed to have non-degradable nature due to hydrophobic backbone Kyaw et al. ( 2012). The synthetic plastics are thus, dumped into landfills or are incinerated. Incineration burns off the plastic waste completely, but at the same time causes heavy toxic fume generation (Al-Salem et al. 2009; Crowley et al. 2003). Recycling is a very environmentally-attractive solution, but a very small part of the plastics can be recycled where the remaining goes to the burial sites (Eili et al. 2012; Bhatia et al. 2013). Thus, there is a need to develop an ‘environment friendly’ degradation solution.

Microorganism – mediated biodegradation of synthetic plastics has been reported to have structural changes particularly with bacteria (Pramila et al. 2012; Kyaw et al. 2012). The most involved bacterial species include *Pseudomonas* and fungal strains are *Aspergillus* and *Penicillium* (Kyaw et al. 2012). The biodegradation is characterized by weight loss (Kyaw et al. 2012), change in mechanical and chemical properties (Roy et al. 2008).

Microorganisms in soil are responsible for degradation, as they utilize hydrocarbons in the polymer backbone as the sole carbon source (Kyaw et al. 2012). Usually, bacterial communities having mixed population are involved, of which the *Pseudomonas* is amongst the extensively found gram negative soil bacterium with the ability to degrade hydrocarbons and various organic molecules (Zhang et al. 2011; Bhattacharya et al. 2003). *Pseudomonas citronellolis* is amongst the polyethylene degrading bacteria (Pramila et al. 2012; Bhattacharya et al. 2003) that belongs to *P. aeruginosa* group (Anzai et al. 2000), it has shown upto 35% of degradation of polyethylene in sample taken from plastic waste dumping site (Shah et al. 2008). The bacteria involve enzymes like monooxigenase, dioxigenase and dehydrogenase to carry out the degradation mechanism (Silva et al. 2010). The enzymes involved cause microbial oxidation (Eubeler et al. 2010) by the capture of oxygen from air as the initial step in biodegradation; further the UV irradiations cause photo-catalytic oxidation (Chiellini et al. 2006) and accelerate the biodegradation process in soil.

The varied bacterial species requires genomic identification to evade the phenotypic identification related problems. (Anzai et al. 2000) Although genomic identification will help in genus identification but the strain still remains unknown for which a phylogenetic assessment is carried out using 16S rRNA sequencing. The 16S rRNA is a universal marker which is used in PCR assay for identification of bacterial species (Rafael et al. 2010).

## Materials and methods

### LDPE Powder preparation

The LDPE sheets were immersed in xylene and boiled for 15 minutes to dissolve completely. The residue obtained was then crushed by hands, wearing gloves. The crushed residue was kept for evaporation and then dried in hot air oven at 60°C overnight. The obtained powder was stored at room temperature in a closed container (Sah et al. 2010).

### Sample collection and screening of LDPE degrading bacteria

The soil sample was collected from Municipal Landfill in Indore. 50 ml of 0.85% saline was prepared and autoclaved, to which 0.1 g collected soil was added in sterile conditions. The solution was kept for incubation at 37°C in a shaker for 4 to 5 hours to be used as inoculum (Burd 2008). The growth medium was prepared by adding 0.1% (NH_4_)_2_ SO_4_, 0.1% NaNO_3_, 0.1% K_2_HPO_4_, 0.1% KCl, 0.02% MgSO_4_ and 0.01% yeast extract in distilled water. 0.2 g of LDPE powder was added to 100 ml of growth medium to prepare the biodegradation medium (Burd 2008).

1% of the prepared inoculum was then transferred to 100 ml of biodegradation medium to isolate the LDPE degrading bacteria and was kept for incubation at 37°C, 200 rpm for 16 to 18 hours. The 1% of the obtained growth was again transferred to 100 ml of biodegradation medium and was kept for incubation. The grown culture was then used as inoculum for nutrient agar plates and was incubated. The mixed colonies were isolated to get pure culture for different isolates. The growth profiles were studied for each of the isolated culture both in presence of 0.2% glucose and 0.2% LDPE as carbon sources in growth medium.

### *In vitro*biodegradation assay

100 ml of growth medium in different flasks was inoculated with the individual obtained bacterial culture and then weighed LDPE sheet pieces were placed in each. LDPE with growth medium and bacterial strain with growth medium were taken as negative and positive controls, respectively. The flasks were then incubated for 24 hours at 37°C, 150 rpm. The OD at 600 nm was recorded after regular intervals of 24 hours till the bacterium reaches stationary phase (Kapri et al. 2010a; Kapri et al. 2009; Sah et al.).

### Recovery of degraded product and sample analysis

The LDPE sheets were recovered after incubation through filtration and were kept for evaporation. The product was then washed using ethanol by centrifugation to remove the bacterial biomass. The obtained product was kept for overnight drying and analysis of recovered LDPE samples was carried out by weight loss percent, SEM, FTIR and TGA (Kapri et al. 2010b; Pramila et al. 2012; Kyaw et al. 2012).

### Bacterial identification

#### Genotypic characterization

The genomic DNA was isolated using phenol\chloroform extraction method. PCR of the isolated genomic DNA was carried out using forward and reverse 16S rRNA primers with DNTP, Buffer and *Taq polymerase*. 30 cycles of PCR were performed and the product was finally stored at 4°C.

The PCR product was run in agarose gel and was amplified using PCR for sequencing. Forward and reverse universal16S rRNA PCR primers were used.

### 16S rRNA Sequencing and Phylogenetic assessment

The product was further sequenced and was subjected to phylogeny assessment. The recently known method that can be used to know the whole genome is next generation sequencing (Nayarisseri et al. 2013) but here only 16S rRNA sequence has been identified to know the strain novelty. The nucleotide sequencing was thus, performed by Applied Bio System Automatic Sequencer Inc., USA. DNA Baser Sequence Assembler v. 1.0 was used to assemble both the forward and reverse sequence file (Anuraj et al. 2012; Shah et al. 2013). The 16S rRNA gene sequences obtained in current study, together with those of *Pseudomonas citronellolis* strain were aligned and sequence similarity was assessed using DNAMan (Phanse et al. 2013). All the related sequences were collected from nucleotide nr database through BLAST. Phylogenetic relationships between *Pseudomonas citronellolis* EMBS027 against other Gram-negative bacterium were inferred from phylogenetic comparison of the 16S rRNA sequences using parsimony (dnapars) and maximum-likelihood algorithms (dnaml and dnamlk) available in Phylip. Maximum likelihood and parsimony-derived trees were bootstrapped using PHYML (Abdennadher & Boesch 2007).

### RNA Structure prediction

The biological function of any system is an outcome of RNA folding. The prediction of RNA secondary structure is based on free energy minimization. The free energy minimization lowers the total Gibbs free energy giving stability to the sequence. RNA structure even helps to determine the evolutionary stability. There are several computational tools in Bioinformatics that generate RNA secondary structures like RNAdraw, RNAfold, UNAFold, etc.

The obtained 16S rRNA sequence was folded using UNAFold to make secondary structure of RNA to check the stability that gives the structure stability in terms of Gibb’s Free Energy (∆G) (Nicholas and Markham 2008).

## Results & discussion

### Screening of LDPE degrading bacterial isolates

**Figure 1:**
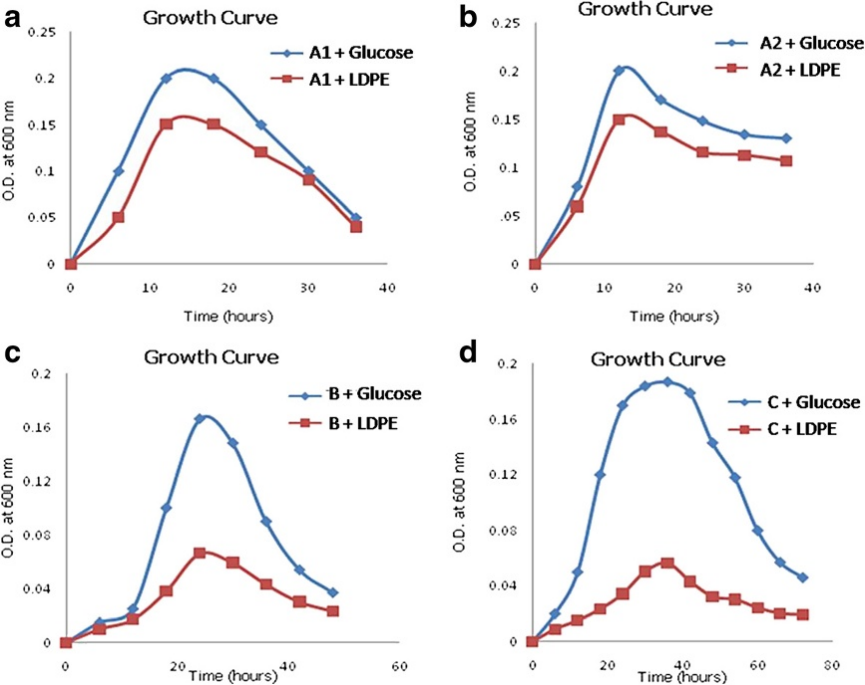
**Growth Profile Study for A1 (a), A2 (b), B (c) and C (d) in presence of Glucose and LDPE as carbon source in growth medium.**

**Figure 2:**
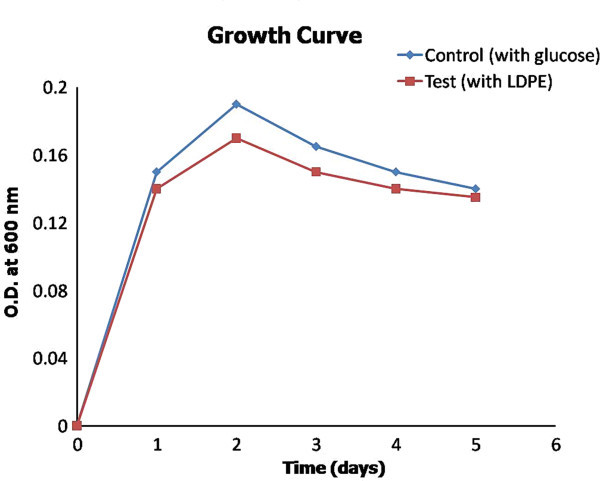
**Growth Profile of Consortium in presence of glucose and LDPE.**

The isolates grown in nutrient broth were transferred to enrichment medium for screening LDPE degrading strains. The cultures obtained in the broth were then grown on nutrient broth. Four isolates were screened with the ability to use LDPE as nutrient medium. A growth profile study of individual strains and culture consortium containing all four isolates was performed by taking OD at regular intervals of 6 hours in presence of glucose and LDPE separately as substrates. Figures 1 and 2 depict the growth curve for individual strains and the consortium, respectively, in presence of glucose and LDPE.

The growth curve formed by consortium was quite supportive of the fact that microbial association is good enough to use the LDPE for cell growth and multiplication. A1 and A2 were having better LDPE assimilation rate than B and C. A1 and A2 were found to give better and efficient in digestion of LDPE in comparison to B and C.

The similar growth profile studies have earlier been reported by Satlewal et al.; Sah et al.; Negi *et al.*; Soni *et al.* to increase microbial biomass by supplementation of different polymers like LDPE, HDPE and epoxy blends (Negi et al. 2009; Negi et al. 2011; Sah et al. 2010a; Satlewal et al. 2008; Soni et al. 2009; Soni et al. 2008). The enzyme secretion by bacterium leads to degradation of substrates like LDPE, HDPE, etc.

### *In vitro*biodegradation assay

**Figure 3:**
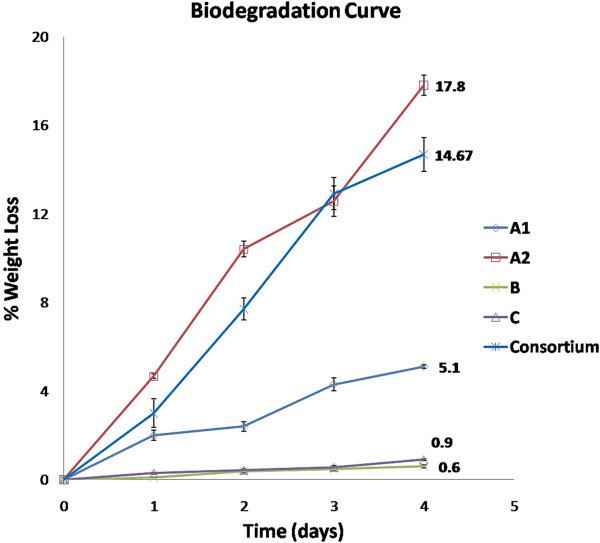
**Growth curve obtained with the biodegradation studies.**

The biodegradation assay was carried out for 96 hours and at regular intervals of 24 hours spectrophotometric data was recorded at 600 nm (Kapri et al. 2010a); Kapri et al. 2009 (Sah et al. 2010). The biodegradation assay was performed with all the four bacterial cultures in growth medium supplemented with LDPE sheets of equal weight. The initial weight and weight at regular intervals, till the cultures reach a stationary phase, for each was recorded. The percent weight loss for each was calculated. The final weight loss of respective cultures has been provided in Table 1 and the graphical study carried out is depicted in Figure 3.

**Table 1:** **Polymer degradation percent achieved by respective bacterium**

	A1	A2	B	C	Consortium
**Weight loss%**	5.1 ± 0.08	17.8 ± 0.45	0.6 ± 0.06	0.9 ± 0.04	14.67 ± 0.75

The maximum degradation was achieved by culture A2 with 17.8% followed by consortium with 14.67% sheet degradation in four days. The potentiality of A1 was quite distinctive than B and C, where former gave 5% and latter has shown weight loss percent of 0.6 and 0.9, respectively.

The culture isolated had efficiency of accumulating on the sheets. Similar biodegradation assay has been documented by Satlewal *et al*. Sah *et al*., Kapri et al. 2010a, Kapri et al. 2010b, Negi *et al*., Soni *et al*. (Negi et al. 2009; Negi et al. 2011; Sah et al. 2010a; Satlewal et al. 2008; Soni et al. 2009; Soni et al. 2008).

The bidegradation assay conducted by Kyaw *et al.* with LDPE treated by *Pseudomonas aeruginosa* was analyzed after *in vivo* study for 120 days that gave 20% weight loss and further the degradation was confirmed by FTIR-ATR spectroscopy (Kyaw et al. 2012). The release of inhibitory enzymes or the competitive action between the secreted enzymes by various culture for single substrate site, might be a reason for lesser degradation by consortium compared to profile as good as A2. Thus based on *in vitro* analysis, A2 was interpretated as LDPE degrading microorganism. Further, characterization study assisted in surface, structure and thermal analysis, leading to identification of LDPE degrading bacterium.

### Characterization of degraded product

#### Surface analysis

**Figure 4:**
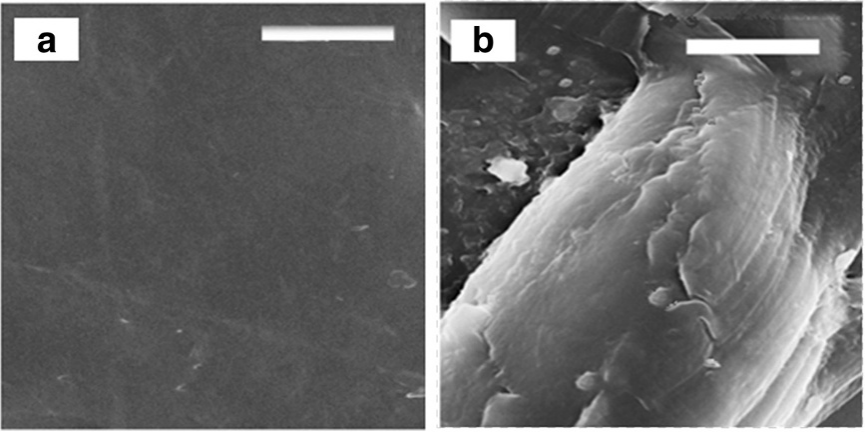
**Scanning Electron Micrographs: (a) Untreated LDPE, (b) Degraded LDPE by A2; Scale = 10 μm, Magnification = 3.00KX.**

Scanning Electron Microscopy of the recovered sample was performed to know the sufacial changes, where pure LDPE was control and the recovered product was test. The SEM micrographs of the undegraded LDPE (Figure 4 (a)) as control illustrated a smooth surface morphology. After 4 days of incubation, the LDPE film degraded by A2 (Figure 4 (b)) was recovered from the biodegradation assay medium.

The worn-out areas with randomly distributed cracks and fissures reveal the disruption of surface texture of LDPE film. However, the extent of biodegradation is identical to the biodegradation efficiency documented by cultures in previously conducted studies by Negi *et al.* (Kapri et al. 2010a; Shrivastav et al. 2011; Negi et al. 2011; Girdhar et al. 2013). Conducted studies on LDPE degradation in presence of bacterial consortium, where the biodegradation assay was followed by LDPE film characterization by SEM lead to capture of cracks and disruption on surface of degraded film in comparison to control (Kapri et al. 2010a; Shrivastav et al. 2011; Negi et al. 2011; Girdhar et al. 2013). Micrographs demonstrates occurrence of several non-uniformlyscattered whitened areas and erosion zones illustrating surface erosion mechnism involved in degradation which might be due to enzyme catalytic action.

### Functional group analysis

**Figure 5:**
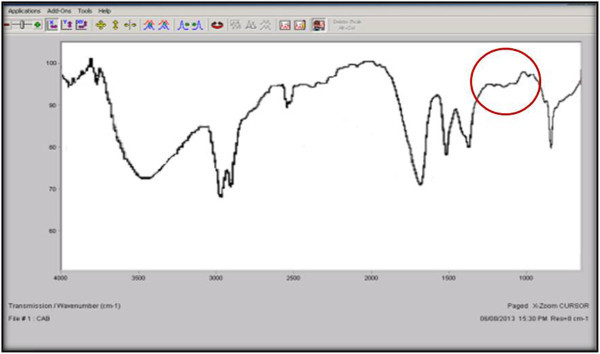
**FTIR spectra of LDPE degraded by A2.**

**Figure 6:**
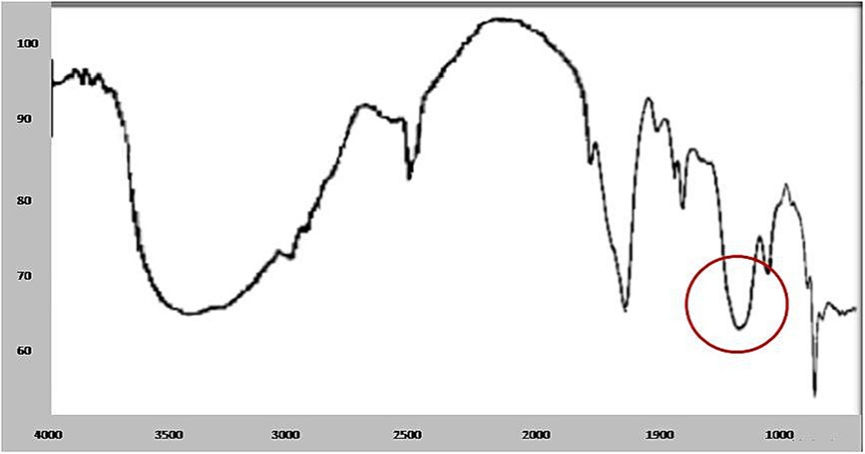
**FTIR spectra of undegraded LDPE (control).**

The structural analysis is an important parameter to know the structural changes appeared due to induced degradation responsible for weight loss. FTIR is sensitive to local molecular environment and as a consequence has been widely applied to investigate the interactions between the macromolecules during degradation. FTIR analysis of the degraded sheet gives a close view of CH stretching at 3,386.9 - 3, 400.9 cm^−1^, CH_2_ deformations of 1,590.6 – 1,595.7 cm^−1^, CH_2_ bending (asymmetrical) at 1,457.3-1,463.6 cm^−1^, and CH_2_ bending (symmetrical) at 1,351.2-1,351.3 cm^−1^ with additional peak at 1128 cm^−1^. The most prominent structural changes were observed in the LDPE sample degraded by A2 bacterial isolate depicted in Figure 5 with the control LDPE peaks (Figure 6).

The LDPE degraded spectra of similar pattern was observed by Sah *et al.* where *Bacillus* and *Arthrobacter* species were implemented for plastic degradation and Negi *et al.* reported a study with *Pseudomonas* and *Microbacterium* species (Sah et al. 2011; Negi et al. 2011). The documented studies have illustrated the polymer degradation by the functional group analysis through FTIR. The addition, deletion and shifting of functional group peaks has been inferred as the major aspect representing the structural changes. The additional peak at 1107.5 cm^−1^ and 1028.1 cm^−1^ were found by Sah *et al.* and Negi *et al.*, respectively when LDPE was treated with consortium and was analyzed after *in vitro* study for 10 days and *in vivo* study for 3 months, respectively. The frequency shifts had been observed due to hydrolysis that led to occurrence of carbonyl group as an additional peak.

### Thermal analysis

**Figure 7:**
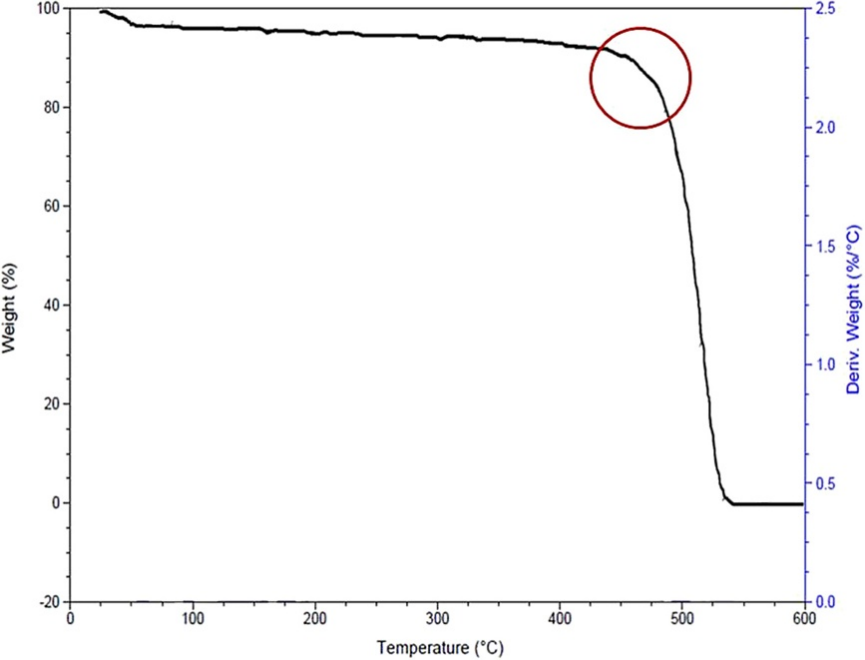
**Thermogravimetric analysis (TGA) of undegraded LDPE (control).**

**Figure 8:**
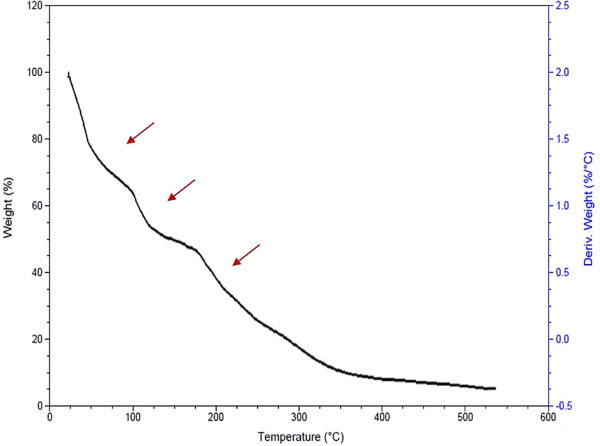
**Thermogravimetric Analysis (TGA) of LDPE degraded by A2.**

Thermal profile of LDPE sheet was checked before and after degradation assay to know the influence of implicating microbial degradation. The thermal profile of undegraded LDPE shows one step steep degradation curve between 450-500°C (Figure 7), where as the thremogravimetric analysis of degraded LDPE shows three step weight loss at 50, 100 and 175°C with weight loss percent of 21.65, 33 and 46, respectively (Figure 8). The results are in line with research conducted by Satlewal *et al*. (Satlewal et al. 2008), according to the documented thermal profile the implication of consortium has shown more than one step degradative mechanism with respect to control i.e. undegraded LDPE and HDPE. With respect to this, in the present study the degradation profile had shown occurrence of similar deformities in the polymer structure, resulting in degradation.

Thus, the study suggests that the indigenous bacterial cultures can accelerate the rate of degradation due to a direct enzymatic scission and assimilation of low-molecular-weight chains that were subsequently being produced due to bacterial digestion of hydrocarbon backbone. The analysis is affirmative of the fact that the bacterium, A2 is degrading LDPE.

### Morphological and biochemical identification of bacteria

The isolated bacterial strains were then characterized based on shape, size, color, opacity, motility and gram’s staining; and the biochemical tests were performed. A1 and A2 have shown maximum degradation in weight loss analysis and were characterized as gram negative short rods with cream colored translucent colonies and motility. The only difference between two were the colony texture were A1 was found to have smooth and A2 had rough colony margins. The biochemical tests even revealed same results for both the strains as oxidase positive, glucose fermentation negative and amino acid utilization positive.

### Genotypic characterization

The genomic DNA was isolated using phenol\chloroform extraction method. PCR of the isolated genomic DNA was performed using forward and reverse 16S rRNA primers with DNTP, Buffer and *Taq polymerase*. A run of 30 PCR cycles was set up with first denaturation step at 95°C for 2 minutes, second step of denaturation at 95°Cfor 30 s (for strand separation), third step was primer annealing conducted at 50°C for 30 s, fourth step is renaturation for 2 minutes at 68°C to built double stranded DNA with *Taq* DNA polymerase and DNTPs, fifth step was extension at 68°C for 10 minutes and the product was finally stored at 4°C.The 16S rRNA primers used were the following forward and reverse sequences, respectively:

27 F : 5' - AGA GTT TGA TCC TGG CTC AG -3'

**Figure 9:**
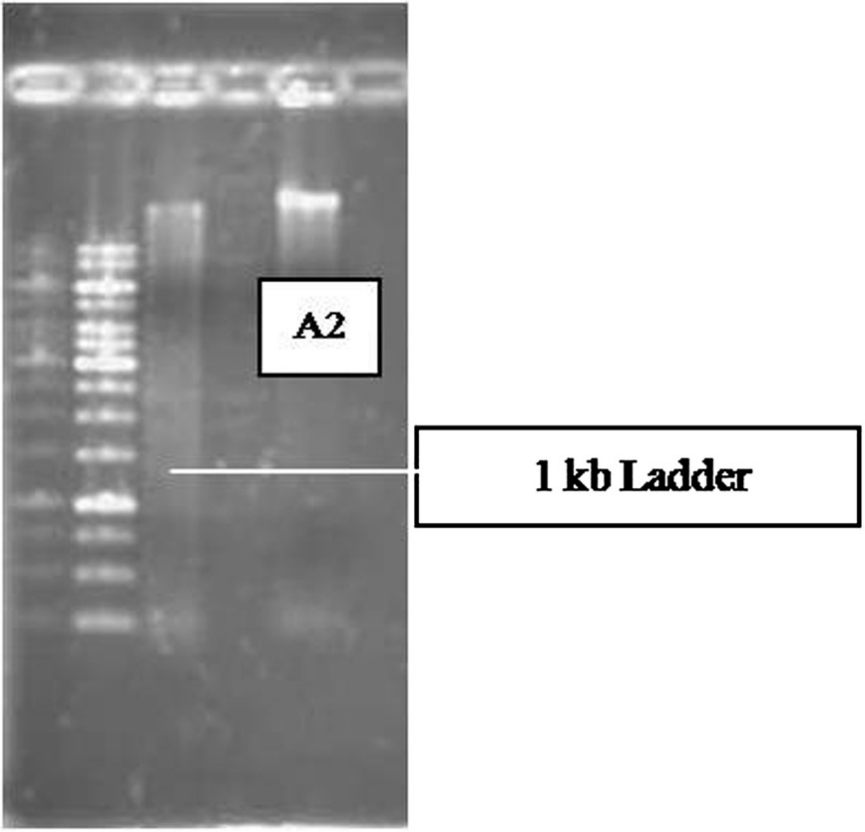
**Genomic DNA isolation.**

**Figure 10:**
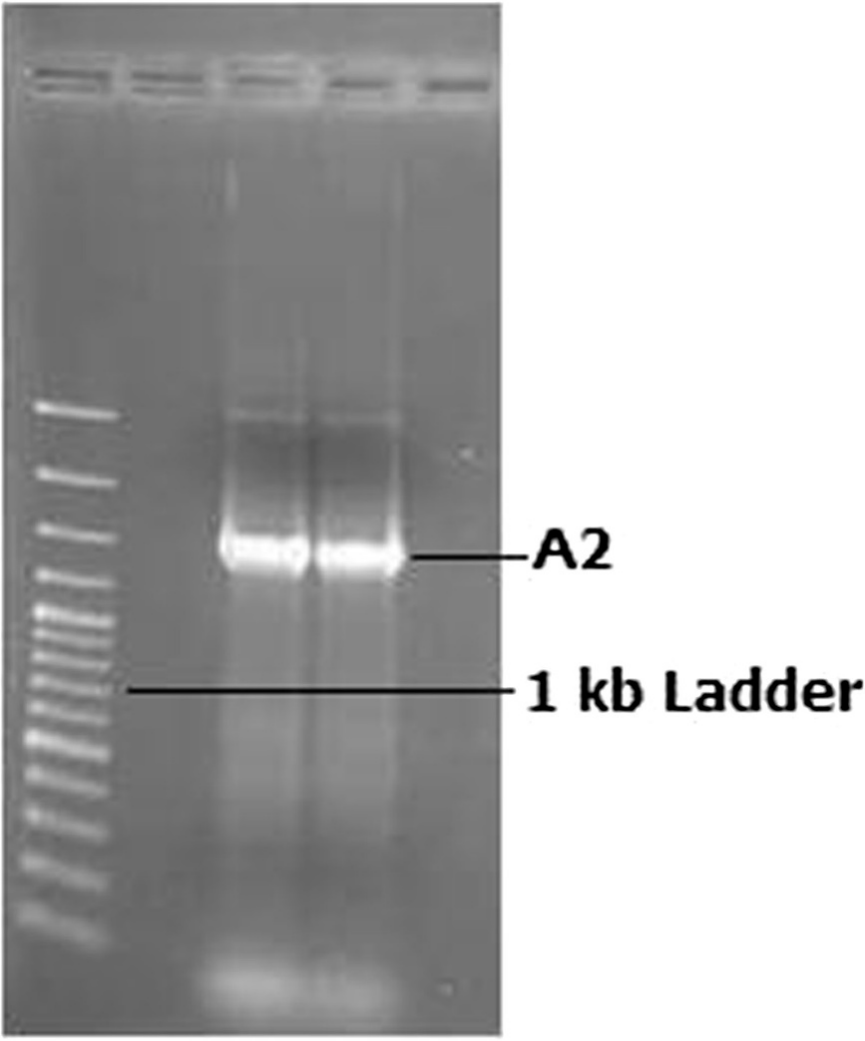
**PCR amplified product.**

1391R : 5' - GAC GGG C(AG)G TG(AT) GT(AG) CA -3'The flanking sites of these primers get annealed and perform amplification of novel bacterial species to know the novel 16S rRNA sequence which is highly conserved but with similar flanking regions. The PCR product was run in agarose gel and the obtained product size was 1.4 kb. The product was further subjected to phylogeny assessment. The genomic DNA after amplication was quantified as 70 mg/ml, at 600 nm OD. Figures 9 and 10 depict the gel pictures of genomic DNA isolation and PCR amplification.

### Sequencing and phylogenetic assessment

The PCR product was subjected to sanger dideoxy sequencing. The sequence thus, obtained was compared against the sequences available in the NCBI, nr database using BLASTn. A2 was found to give 96% similarity to the existing *Pseudomonas citronellolis.*

**Figure 11:**
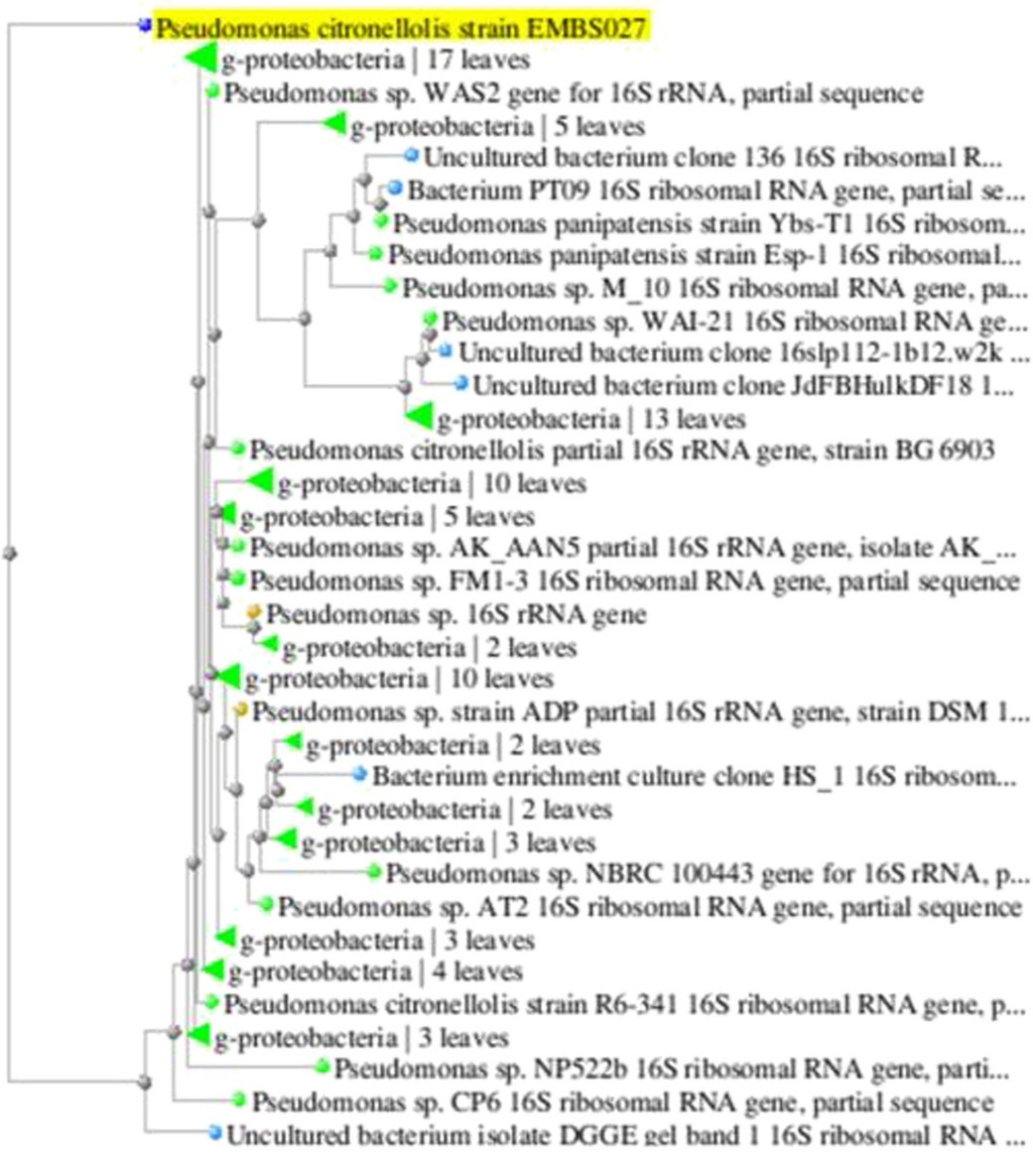
**Phylogenetic relation of Pseudomonas citronellolis strain EMBS027 against other species.**

DNA Baser Sequence Assembler v. 1.0 was used to assemble both the forward and reverse sequence file (Anuraj et al. 2012; Shah et al. 2013). The 16S rRNA gene sequences obtained in current study, together with those of *Pseudomonas citronellolis* strain were aligned and sequence similarity was assessed using DNAMan (Phanse et al. 2013). Phylogenetic relationships between *Pseudomonas citronellolis* EMBS027 against other bacteria was inferred from phylogenetic comparison of the 16S rRNA sequences using parsimony (dnapars) and maximum-likelihood algorithms (dnaml and dnamlk) available in Phylip. Maximum likelihood and parsimony-derived trees were bootstrapped using PHYML (Abdennadher & Boesch 2007). Figure 11 shows phylogenetic tree.

The result of phylogenetic analysis revealed A2 bacterium as a novel LDPE degrading *Pseudomonas* species, which was further named as *Pseudomonas citronellolis* EMBS027. After characterization the sequence of isolate was deposited in GenBank with Accession number KF361478, maintained by the National Centre for Biotechnology Information (NCBI), at National Institute of Health (NIH), Rockville, Maryland, US.

The obtained sequence of Pseudomonas citronellolis EMBS027 contains 1476 base pairs with Molecular Weight for single stranded and double stranded as 447604.00 Daltons and 898163.00 Daltons, respectively. GC content was 53.46% and AT content was 46.54%.

### RNA Structure prediction

The RNA structure of the sequence was predicted using UNAFold to know the stability of gene sequence. The stability was calculated as Gibb’s free energy. Additional file 1 shows the structure. The value obtained for ∆G = −517.73 kcal/mol.

The present study has employed UNAFold which is an advanced version based on the earlier used mFold tool. UNAFold uses nearest neighbor energy rule to calculate the energy of the structure. Singh *et al.* predicted *A. veronii AV25* RNA structure with Gibb’s free energy of −322.40 kcal/mol ( Hooker & Rosulek 2010; Singh et al. 2012). In the present modeled structure gibbs free energy specifies the fold stability and also provide energy minimized structure, but can deviate in natural complexities of the system. The prediction is a proof of the stability of nucleotides in the novel *Pseudomonas citronellolis* EMBS027, though can be used for extracting useful information when implicated in future studies.

The biodegradation study has characterized A2 as LDPE degrading strain. Further, phylogenetic assessment has specified A2 strain as novel among the *Pseudomonas citronellolis* strains, and has been avowed as *Pseudomonas citronellolis* EMBS027.

## Electronic supplementary material

Additional file 1: **RNA secondary structure.** (PDF 263 KB)
