# Supplementary figures and images for: Implications of a novel Pseudomonas species on low density polyethylene biodegradation: an in vitro to in silico approach
Source: Springerplus. 2014 Sep 2;3:497. doi: 10.1186/2193-1801-3-497 (PMC4409612; doi:10.1186/2193-1801-3-497)

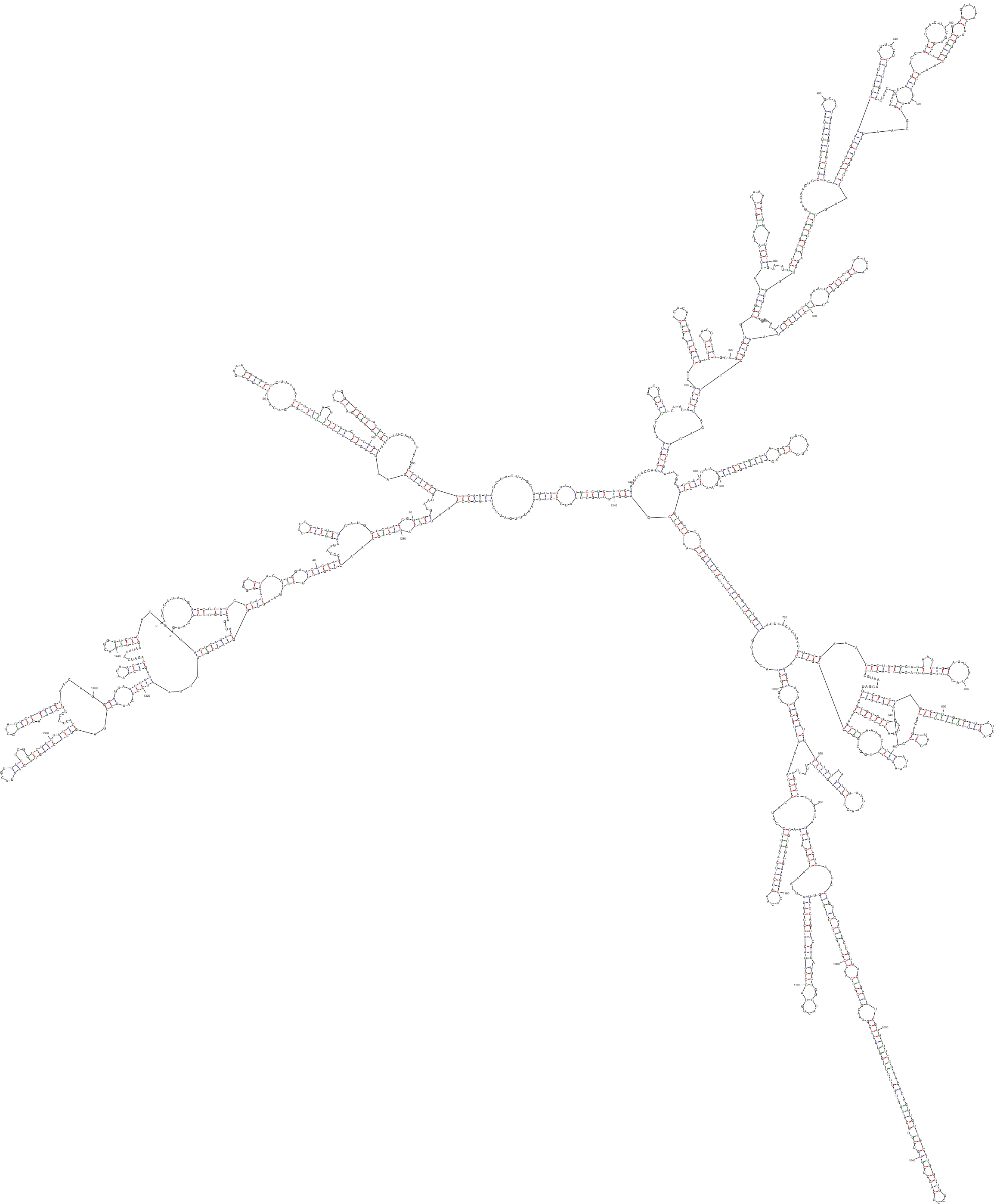

Supplement: Supplementary file 1 — Additional file 1: RNA secondary structure. (PDF 263 KB) [file 40064_2014_1478_MOESM1_ESM.pdf]
